# Pan-Cancer Analysis of PARP1 Alterations as Biomarkers in the Prediction of Immunotherapeutic Effects and the Association of Its Expression Levels and Immunotherapy Signatures

**DOI:** 10.3389/fimmu.2021.721030

**Published:** 2021-08-31

**Authors:** Xinke Zhang, Yingying Wang, Gari A, Chunhua Qu, Jiewei Chen

**Affiliations:** ^1^State Key Laboratory of Oncology in South China, Collaborative Innovation Center for Cancer Medicine, Sun Yat-sen University Cancer Center, Guangzhou, China; ^2^Department of Pathology, Sun Yat-sen University Cancer Center, Guangzhou, China; ^3^Department of Scientific Research Management, Affiliated Cancer Hospital and Institute of Guangzhou Medical University, Guangzhou, China

**Keywords:** pan-cancer, PARP1, immunotherapeutic, biomarkers, immunotherapy signatures

## Abstract

**Background:**

Poly (ADP-ribose) polymerases-1 (PARP1) alterations are associated with PARP1 inhibitor resistance, regulating the function of Treg cells and PDL1 expression in tumor cells, and high PARP1 expression is significantly associated with aggressive behavior and chemotherapeutic resistance in several tumors. However, a comprehensive analysis of the predictive values of PARP1 alteration for immune checkpoint inhibitor (ICI) effectiveness in tumors remains unclear, and the associations between its expression and immunotherapy signatures also needs to be explored further.

**Methods:**

We performed some analyses with the cBioPortal online database (https://www.cbioportal.org), TIMER2.0 (Tumor Immune Estimation Resource 2.0, http://timer.comp-genomics.org/) and TCGA database (https://xenabrowser.net or https://portal.gdc.cancer.gov/). Survival analysis was conducted using Kaplan–Meier method, and the associations between PARP1 transcription levels and immune checkpoint gene expression, the number of neoantigens, tumor mutation burden (TMB) levels, and microsatellite instability (MSI) event are analyzed by spearman correlation analysis and visualization of those mentioned above is performed using R, version 3.6.3 (http://www.r-project.org/).

**Results:**

We found that PARP1 was altered in 1338 (2.9%) out of 45604 patients with diverse tumors, which was associated with markedly higher TMB levels in a variety of tumors (P < 0.01). Impressively, patients with PARP1 alterations in advanced tumors showed better overall survival (OS) in the ICI-treated cohort (P = 0.016). PARP1 altered group was substantially correlated with higher immune infiltrates across most tumors, including CD8+ T cells in colorectal adenocarcinoma (P = 0.0061), endometrial carcinoma (P = 0.0033), stomach cancer (P = 0.033), and cervical cancer (P = 0.026), respectively. The PARP1 altered group showed high expression in transcription (P < 0.001), and higher expression of LAG3, PDCD1, CTLA-4, and TIGIT (P < 0.05). Higher PARP1 expression was present in 27 tumor compared the corresponding normal tissues using the GTEx and TCGA databases and it had a worse OS in several tumors (P < 0.05). Further, high PARP1 expression was significantly associated with six immune cells (B cells, CD4+ T cells, CD8+ T cells, macrophages, neutrophils, and dendritic cells) in most tumors, including colon adenocarcinoma (COAD), head and neck squamous cell carcinoma (HNSC), kidney renal clear cell carcinoma (KIRC), and liver hepatocellular carcinoma (LIHC) (P < 0.05). In particular, CD8+T cell infiltration, was also positively correlated with high PARP1 expression in bladder urothelial carcinoma (BLCA), breast invasive carcinoma (BRCA), kidney renal papillary cell carcinoma (KIRP), brain lower grade glioma (LGG), LIHC, pancreatic adenocarcinoma (PAAD), pheochromocytoma and paraganglioma (PCPG), prostate adenocarcinoma (PRAD), rectum adenocarcinoma (READ), testicular germ cell tumors (TGCT), thymoma (THYM), uterine corpus endometrial carcinoma (UCEC), uveal melanoma (UVM) (P < 0.05, no data shown), and PARP1 expression was significantly positively correlated with the transcription levels of some of the 47 immune checkpoint genes, such as CD274, CTLA4, and PDCD1 in several tumors, including PAAD, LIHC, KIRC, HNSC, and BLCA (P < 0.05). A significant positive association between PARP1 expression and the number of immune neoantigen was found within COAD, KIRC, lung adenocarcinoma (LUAD), PAAD and THYM (P < 0.05), and there were also significantly positive correlations between PARP1 expression and TMB in many tumors like adrenocortical carcinoma (ACC), COAD, kidney chromophobe (KICH), LGG, LUAD, READ, skin cutaneous melanoma (SKCM) and stomach adenocarcinoma (STAD) (P < 0.05). In addition, high PARP1 expression was positively associated with microsatellite instability event in COAD, KIRP, BRCA, glioblastoma multiforme (GBM), lung squamous cell carcinoma (LUSC), LGG, READ, UCEC, SKCM and LUAD (P < 0.05).

**Conclusions:**

Our results highlight the significance of PARP1 alterations as pan-cancer predictive biomarkers for ICI treatment, and its expression levels seem to be correlated with the status of immunotherapy-associated signatures, thus may be a promising biomarker for predicting ICI response in several tumors.

## Introduction

Novel immune checkpoint inhibitors (ICIs), including anti-cytotoxic T lymphocyte antigen 4 (CTLA-4), programmed death 1 (PD-1), and their ligands, PDL1 (CD274) and PD-L2 (CD273), have great potential for therapeutic efficacy in a variety of tumors, such as metastatic melanoma and non-small cell lung carcinoma (NSCLC), along with bladder urothelial cancer (1). However, patients treated with ICIs have variable response rates, and in several cases, a low treatment response rate has limited their practical application (2, 3). Therefore, current research is focused on how to predict patients who are responders and non-responders to ICI treatment (4). With our continual improvement in understanding genomic and microenvironmental processes associated with responses to ICIs, it becomes more beneficial for us to find biomarkers that could predict treatment effectiveness to ICIs, and it had also advantage in terms of supporting selection of patients and decision making by separating responders and non-responders accordingly (5, 6).

Poly (ADP-ribose) polymerases-1 (PARP1), a member of the PARP family, was identified as a DNA damage sensor that plays a critical role in the process of DNA damage repair, along with regulation of the expression of genes by poly(ADP-ribosyl), which is a sequence-specific DNA-binding transcription factor (7). Mutations in PARP1 have been found in several tumors, and some point mutations are closely linked to PARP1 inhibitor resistance (8). A recent study reported that PARP1 could ADP-ribosylate the regulatory T-cell (Treg)-specific transcription factor FOXP3, that negatively regulates the function of Treg cells, and PARP1 silencing can enhance PDL1 expression in tumor cells (9, 10). In preclinical experiments on breast cancer cell lines, small-molecule PARP1/PD-L1 inhibitor conjugates demonstrated more significant apoptosis and cytotoxic efficacy compared to their separate agents (11). These results suggest that not only could PARP1 alteration incorporate ICI treatment but also seem to potentially predict ICI therapeutic values. However, a systematic analysis of the prevalence and predictive value of PARP1 alteration for ICI therapeutic effectiveness in multiple tumor types remains largely unknown, and the associations between its expression levels and immunotherapy-associated signatures also need to be explored.

In our pan-cancer analysis study, we comprehensively investigated the frequency of PARP1 alterations and their predictive value in a variety of tumor types through an online database. There has been a frequency (2.9%) in PARP1 alterations and a significant predictive value for ICI treatments across more than 40,000 patients with various tumors. We analyzed the relationship between PARP1 alterations and tumor mutation burden (TMB) level, immune cell infiltrations, along with microsatellite instability (MSI) event. PARP1 alterations could predict the effectiveness of ICI therapies, as evidenced by our analysis results. Meanwhile, we performed a relationship analysis of PARP1 expression with TMB, MSI, and tumor immune infiltrations as well, based on the online database.

## Materials and Methods

### cBioPortal

The data in cBioPortal (https://www.cbioportal.org) is derived from the International Cancer Genome Consortium (ICGC), TCGA, GEO, and other databases, which includes DNA methylation data, limited clinical data mRNA and microRNA expression data, non−synonymous mutations, protein level and phosphoprotein level [reverse-phase protein array (RPPA)] data, and DNA copy number data (12). Clinical and sequencing data of patients were downloaded from the cBioPortal online database. We utilized these data to analyze the rate of PARP1 alterations and the association between its alteration and TMB, MSI, and prognosis. Tumor mutation burden (TMB) is usually measured by the number of somatic mutations (non-synonymous mutations) in the coding region (exon region) of the tumor cell genome. The types of mutations mainly include single nucleotide variation (SNV), and the insertion/deletion of small fragments. TMB is used to reflect the number of mutations within tumor cells and is a quantifiable biomarker, which is the latest for evaluating the therapeutic effect of PD-1 antibodies (13). Microsatellite Instability (MSI), compared with normal tissues, a new microsatellite allele appears at a certain microsatellite site in a tumor due to the insertion or deletion of repeat units. The occurrence of MSI is caused by functional defects in the repair of DNA mismatches in tumor tissues. The MSI phenomenon accompanied by DNA mismatch repair defects is an important tumor marker. PD-1 antibody has been proven for the treatment of tumors with mismatch repair defects (14).

### TIMER2.0

TIMER2.0 (http://timer.comp-genomics.org/) is a tumor immunity related database. “Immune association module” refers to analyzing the association between gene expression, mutation status, somatic CNV and immune cell types. “Immune estimation module” refers to analyzing immune infiltration estimations for users-provided expression profiles by TIMER, CIBERSORT, quanTIseq, xCell, MCP-counter and EPIC algorithms (15). The estimation of immune infiltration in tumors with or without PARP1 alterations by users-provided expression profiles CIBERSORT algorithms to learn about the differences in multiple immune cell types, or PARP1 expression by users-provided expression profiles TIMER algorithms only to learn about the differences in six immune cell types was performed using TIMER2.0 (Tumor Immune Estimation Resource 2.0, http://timer.comp-genomics.org/). Immune infiltration cells include lymphocytes, macrophages, NK cells and neutrophils, etc., which can effectively predict the prognosis of patients (16). We have studied whether the expression of PARP1 gene is related to the level of immune infiltration of different tumor types. The scores of six immune infiltrating cells of 33 tumors were obtained in the TIMER2.0 database, and the correlation between PARP1 gene expression and these immune infiltrating cells was analyzed.

### TCGA Database

(https://xenabrowser.net or https://portal.gdc.cancer.gov/).

For pan-cancer data of TCGA and GTEx databases, differential PARP1 mRNA expressions between various tumors and normal tissues were analyzed using T-test and their visualization was carried out by R software package “ggplot2”. Survival curves of PARP1 mRNA expressions using the optimal cutoffs and overall survival in different tumors were performed by R software package”survminer”. We used the R software package “estimate” to analyze the immune scores and stromal scores in various tumors, and visualized the correlation between PARP1 gene expression and these scores in 33 tumors. The correlation heatmaps between PARP1 expression and antigen presenting molecule, immune checkpoint genes were plotted using R software package “corrplot”. We used TCGA WES data to calculate the TMB levels in a diversity of tumors by R software package “maftools” and visualization of association between PARP1 expression and TMB levels was performed by Radar chart. Finally, we downloaded the data of different tumors involved neoantigens (17) and MSI event (18) from PUBMED (https://pubmed.ncbi.nlm.nih.gov/) and visualization of association between PARP1 expression and MSI event, neoantigens was performed by Radar chart and scatter plot, respectively.

### Statistical Analysis

Survival analysis was conducted using Kaplan–Meier method, and the associations between PARP1 transcription levels and immune checkpoint gene expression, the number of neoantigens, TMB levels, and MSI status are analyzed by spearman correlation analysis and visualization of those mentioned above is performed using R, version 3.6.3 (http://www.r-project.org/). A two-tailed P-value less than 0.05 was defined as statistically significant.

## Results

### PARP1 Alterations and Their Relationship With TMB Level and MSI Status

**Figure 1:**
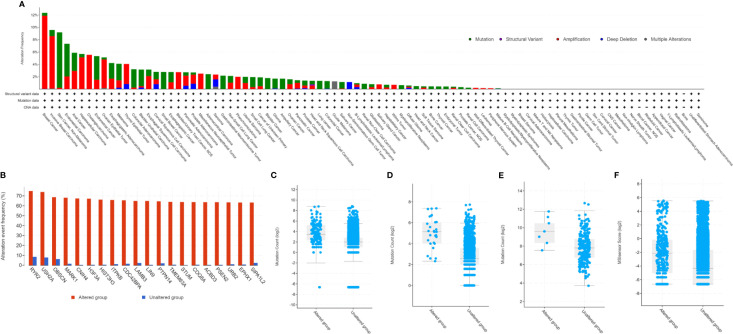
Genetic alterations of PARP1 and its association with TMB level. Prevalence of PARP1 alterations in variety of tumor types **(A)**; Co-occurrence of genetic mutations in tumors with PARP1 alterations **(B)**; The association between TMB and PARP1 alterations in MSK-IMPACT cohort **(C)**, immune checkpoint inhibitors treatment cohort **(D)**, and microsatellite-stable solid tumors received immune checkpoint inhibitors treatment **(E)**; The association between MSI status and PARP1 alternations in TCGA pan-caner cohorts **(F)**.

Our study found PARP1 alterations as a variety of nonsynonymous mutations, including missense, frameshift, splice site, nonsense, fusions, inframe, and deletions and amplification. Cbioportal showed that the PARP1 gene was altered in 1338 (2.9%) of 45604 patients with diverse tumors. Patients with breast cancer had the highest frequency of PARP1 alterations (12.35%), and most of the alterations were amplification (11.82%, 545/4609, **Figure 1A**). However, patients with skin cancer and non-melanoma had the highest mutation frequency (8.98%, 44/490, **Figure 1A**), followed by those with endometrial carcinoma (5.29%, 31/586, **Figure 1A**). There was a different landscape and frequency of PARP1 alterations in early-stage (TCGA cohort; **Supplementary Figure S1A**) and advanced-stage tumors (MSK-IMPACT cohort; **Supplementary Figure S1B**). Co-occurrence of genetic mutations in tumors with PARP1 alterations involved some genes that were enriched in transcriptional misregulation in cancer pathways (e.g., H3F3A, HIST3H3) and calcium signaling pathways (e.g., ITPKB, RYR2) (**Figure 1B**). Compared to advanced-stage tumors (MSK-IMPACT cohort; **Supplementary Figure S2B**), there was a different co-occurrence spectrum of genetic mutations observed in early-stage (TCGA cohort; **Supplementary Figure S2A**) tumors with PARP1 alterations. Then, we analyzed TMB levels of patients with PARP1 altered and unaltered groups in a subset of the MSK-IMPACT clinical sequencing cohort from the TCGA database, in which 149 (1.4%) of 10336 patients had PARP1 alterations, with TMB levels in the PARP1 altered group being significantly elevated compared to the unaltered group (median value: 11 *vs.* 4 mutations/Mb, P < 0.0001; **Figure 1C**). Similar results were observed in two ICI-treated cohorts (P < 0.0001 and P = 0.0054; **Figures 1D, E**). Although rare multiple PARP1 alterations were found, TMB levels were higher than those in other alternations (**Supplementary Figures S2C, D**). Finally, we found that PARP1 alterations were markedly associated with MSI status in TCGA pan-cancer cohorts (P < 0.0001; **Figure 1F**).

### Prognostic and Predictive Value of PARP1 Alterations

**Figure 2:**
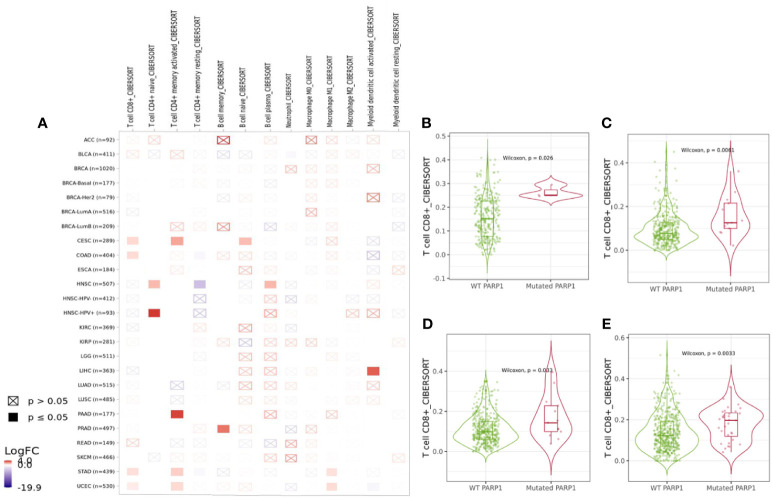
Prognostic and predictive value of PARP1 alterations. Survival analysis between PARP1 alterations and overall survival in the whole tumors **(A)**; Survival analysis between PARP1 alterations and progress-free survival in the whole tumors **(B)**; Predictive value of PARP1 alterations in patients received ICIs treatment **(C)**; Subgroup analysis the predictive value of PARP1 alterations subtypes in patients received ICIs treatment **(D)**. The association between clinical benefit and PARP1 alterations **(E)**.

Among all the groups, when comparing with the PARP1 unaltered group, we initially found that the PARP1 altered group had a significantly longer overall survival (P < 0.0001; **Figure 2A**), and there was a statistical tendency towards a longer progression-free survival (P = 0.11; **Figure 2B**) in the PARP1 altered group than in that without the alteration. In early-stage tumors, the PARP1 altered group was associated with longer OS (P = 0.044; **Supplementary Figure S3A**), and there was also a statistical tendency towards a prolonged PFS and DFS (P = 0.120, P = 0.110; **Supplementary Figures S3B, C**). The prognostic value of PARP1 alterations was not observed in advanced-stage tumors around OS (P = 0.470) (**Supplementary Figure S3D**). In the ICI therapy cohort, 28 (2%) of the 1661 patients with various tumors had PARP1 alterations. Patients with PARP1 alterations had a better OS than those in the unaltered group (P = 0.016; **Figure 2C**), and the multiple PARP1 altered group had the best OS compared to those with single PARP1 altered or unaltered in the subgroup analysis (P = 0.047; **Figure 2D**). In ICI-treated cohorts, PARP1 alterations were somewhat linked to a better ICI treatment effect than in the unaltered group (42.9% *vs.* 27.7%, P = 0.376; **Figure 2E**). However, the PARP1 altered group was not closely associated with OS and DFS in patients with microsatellite-stable (MSS) solid tumors (P = 0.21, p = 0.36; **Supplementary Figures S4A-B**).

### Association of PARP1 Alterations With Immune Checkpoints and Immune Cells

**Figure 3:**
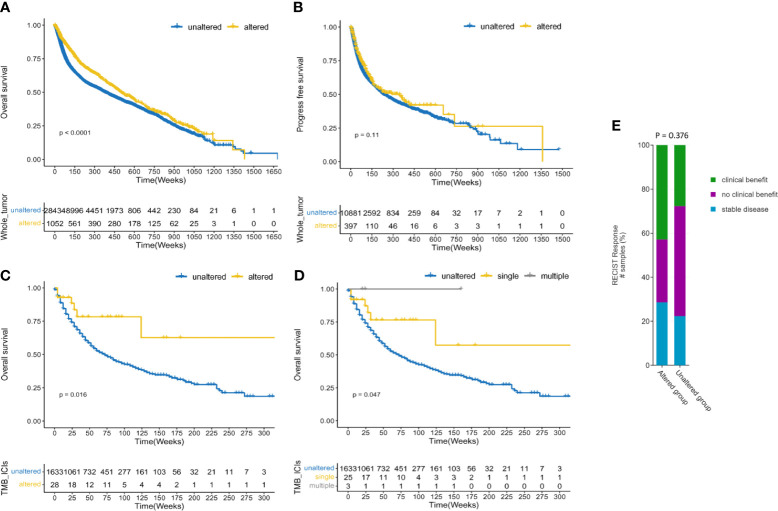
Immune landscape of tumors with PARP1 alterations. The association between PARP1 alterations and immune infiltrates in different tumors **(A)**; Cervical cancer **(B)**; Colon adenocarcinoma **(C)**; Stomach carcinoma **(D)**; Endometrial cancer **(E)**.

We found that PARP1 altered group was significantly associated with higher expression of LAG3, PDCD1 (P<0.01), CTLA-4 and TIGIT (P<0.05) in transcription levels than unaltered group (**Supplementary Figure S5A**). The association between PARP1 alterations and immune infiltrates in different tumors was showed in **Figure 3A**, and then PARP1 altered group was substantially correlated with higher immune infiltrates across several tumors, including CD8+ T cells in colorectal adenocarcinoma (P = 0.0061), endometrial carcinoma (P = 0.0033), stomach cancer (P = 0.033), and cervical cancer (P = 0.026) (**Figures 3B–E**). As yet, comparing with unaltered group, there was lower level of CD8+ T cells in PARP1 altered group in head and neck squamous cell carcinoma. Copy number variations (either deletion or amplification) of PARP1 were significantly linked with lower six types of immune infiltrates in many tumors like head and neck squamous cell carcinoma, kidney renal clear cell carcinoma, lung adenocarcinoma, pancreatic adenocarcinoma, skin cutaneous melanoma and endometrial cancer (**Supplementary Figures S5C-H**).

### PARP1 Expression in Tumors and Its Association With Immune Checkpoints and Immune Cells

**Figure 4:**
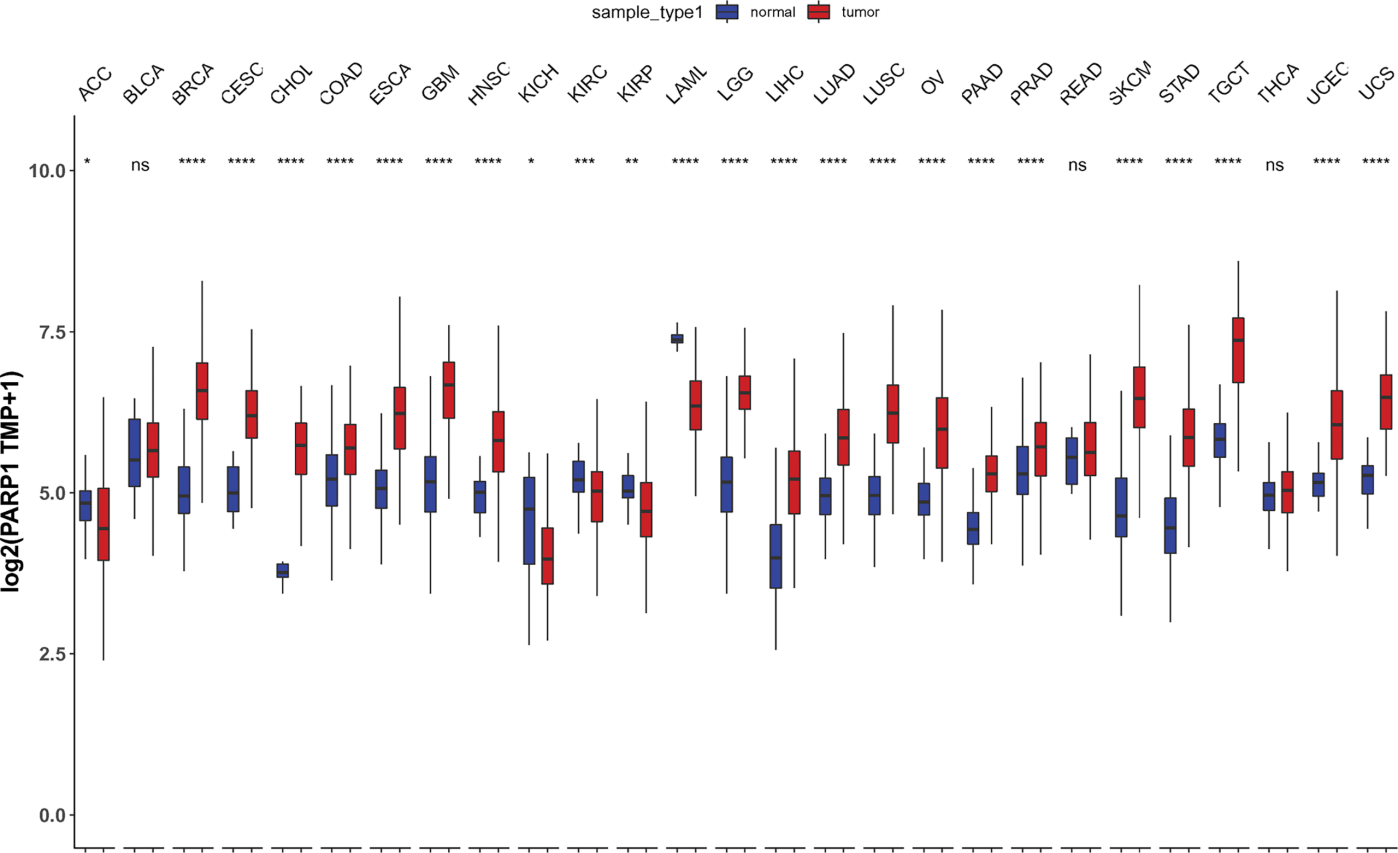
Differential PARP1 mRNA expression in 27 tumors by TCGA database integrating the normal tissue data in the GTEx. *p < 0.05; **p < 0.01; ***p < 0.001; ****p < 0.0001; ns, p > 0.05.

**Figure 5:**
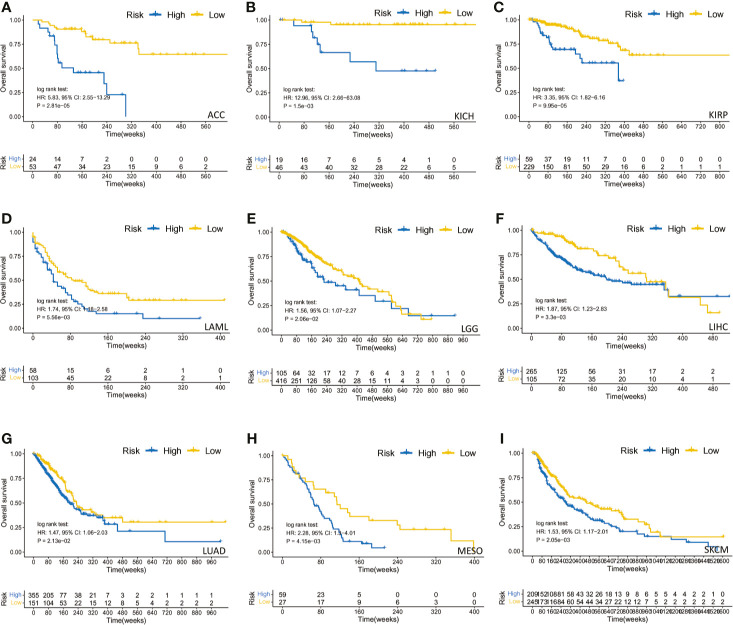
The survival analysis of PARP1 expression and overall survival in several tumors. ACC **(A)**; KICH **(B)**; KIRP **(C)**; LAML **(D)**; LGG **(E)**; LIHC **(F)**; LUAD **(G)**; MESO **(H)**; SKCM **(I)**.

**Figure 6:**
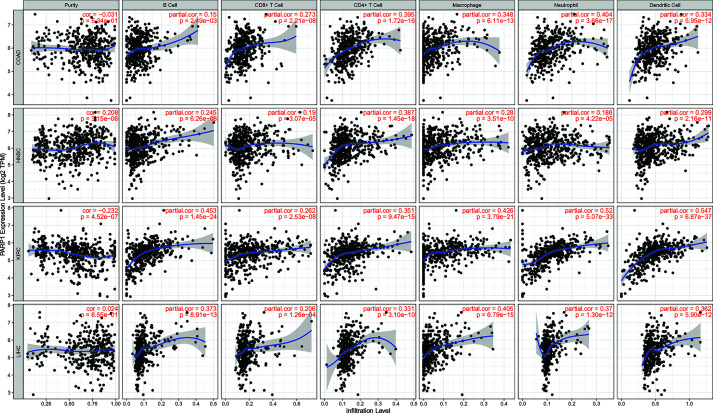
The association between PARP1 expression and immune cell infiltrations in top 4 tumors. COAD, HNSC, KIRC and LIHC (TIMER database showed).

**Figure 7:**
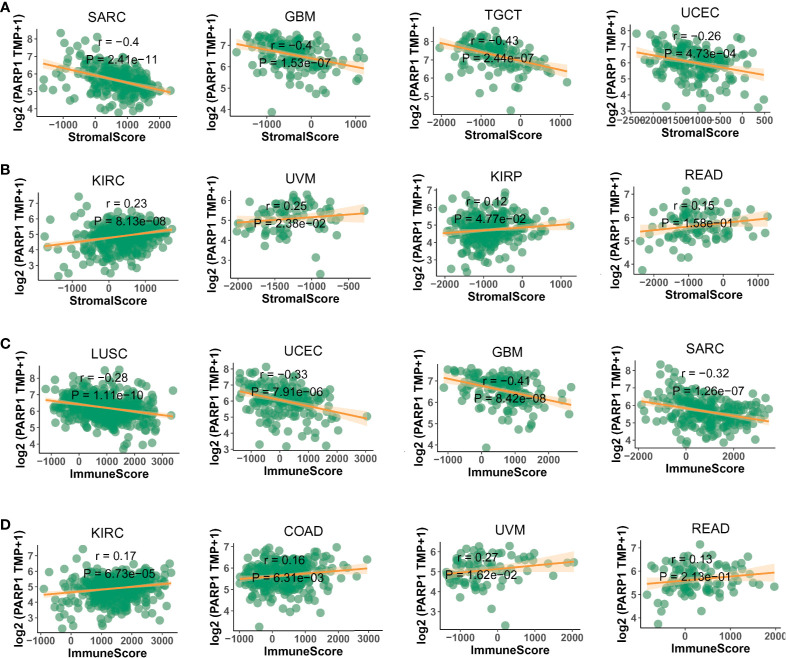
The negative or positive association between PARP1 expression and stromal score **(A, B)** and immune score **(C, D)** in top 4 tumors.

**Figure 8:**
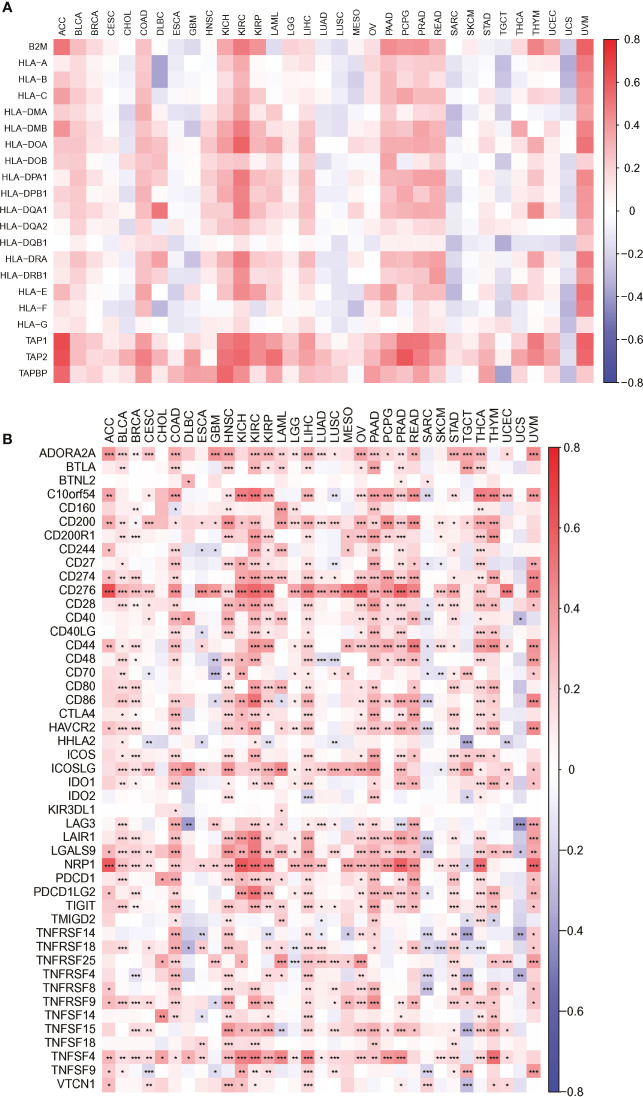
The association heatmaps between PARP1 expression and antigen presenting molecular levels **(A)**, immune checkpoints genes expression in 33 tumors **(B)**. *P < 0.05, **P < 0.01, ***P < 0.001.

We also found that most tumors had high expression of PARP1 mRNA, compared to corresponding normal tissues (**Figure 4**), and there was a worse OS in patients with high expression of PARP1 mRNA for several tumors (**Figure 5**). TIMER data showed high PARP1 expression was significantly associated with six immune cells (B cells, CD4+ T cells, CD8+ T cells, macrophages, neutrophils, and dendritic cells) in most tumors, including colon adenocarcinoma (COAD), head and neck squamous cell carcinoma (HNSC), kidney renal clear cell carcinoma (KIRC), and liver hepatocellular carcinoma (LIHC) (**Figure 6**). In particular, CD8+T cell infiltration, was also positively correlated with high PARP1 expression in bladder urothelial carcinoma (BLCA), breast invasive carcinoma (BRCA), kidney renal papillary cell carcinoma (KIRP), brain lower grade glioma (LGG), LIHC, pancreatic adenocarcinoma (PAAD), pheochromocytoma and paraganglioma (PCPG), prostate adenocarcinoma (PRAD), rectum adenocarcinoma (READ), testicular germ cell tumors (TGCT), thymoma (THYM), uterine corpus endometrial carcinoma (UCEC), uveal melanoma (UVM) (P < 0.05, no data shown). High PARP1 expression was positively associated with higher immune score and stromal score in KIRC, COAD, UVM, KIRP and READ (**Figure 7**). In addition, there were distinctly negative associations between PARP1 expression and human leukocyte antigen (HLA)-I and II molecular levels in several tumors (**Figure 8A**). PARP1 expression was significantly positively correlated with the transcription levels of some immune checkpoint genes (19), such as CD274, CTLA4, and PDCD1, in several tumors including PAAD, LIHC, KIRC, BLCA, and HNSC (**Figure 8B**).

### The Relationships Between PARP1 Expression and Immune Neoantigen, TMB, and Microsatellite Instability Event

**Figure 9:**
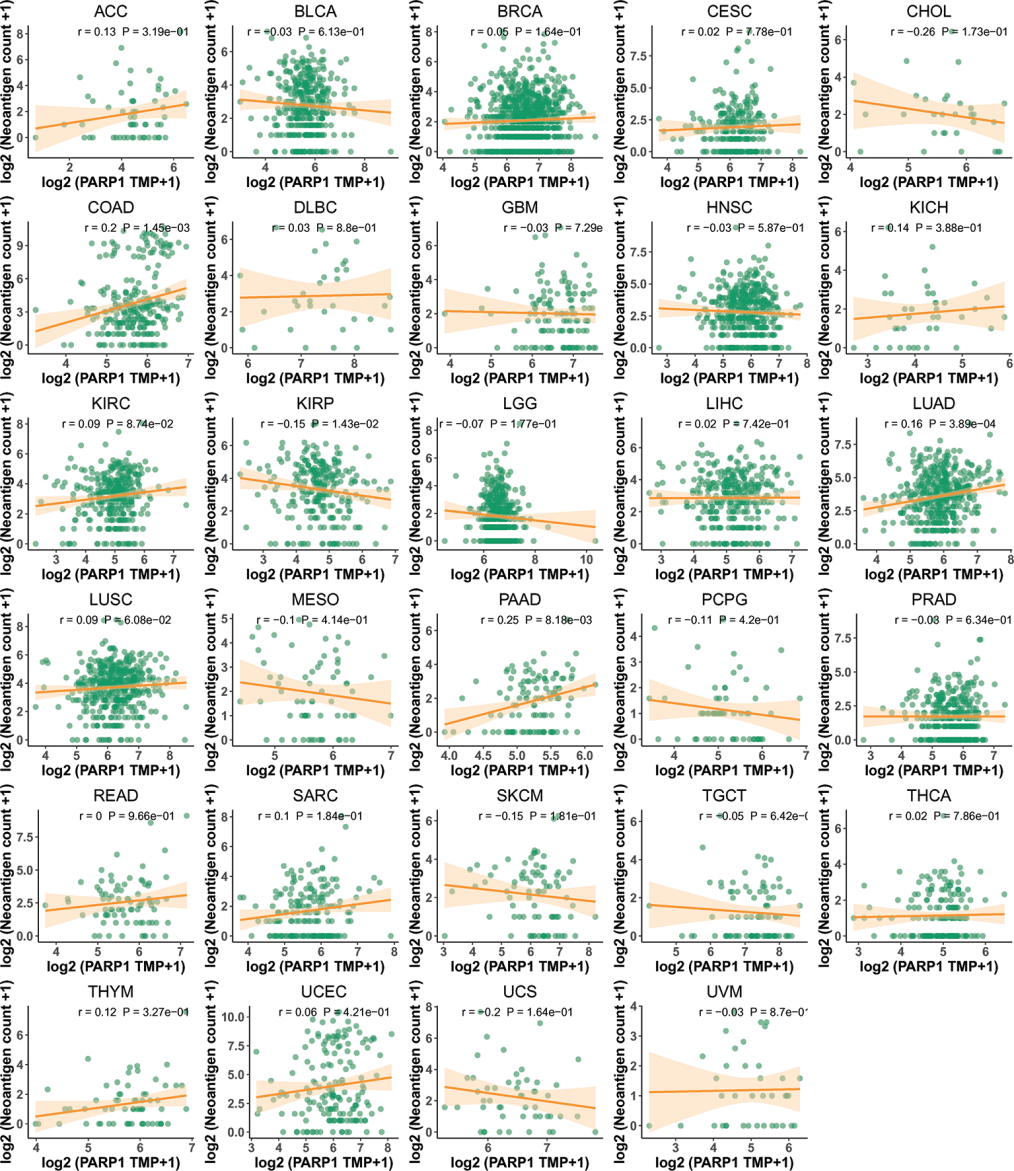
The association between PARP1 expression and the number of neoantigen in tumors.

**Figure 10:**
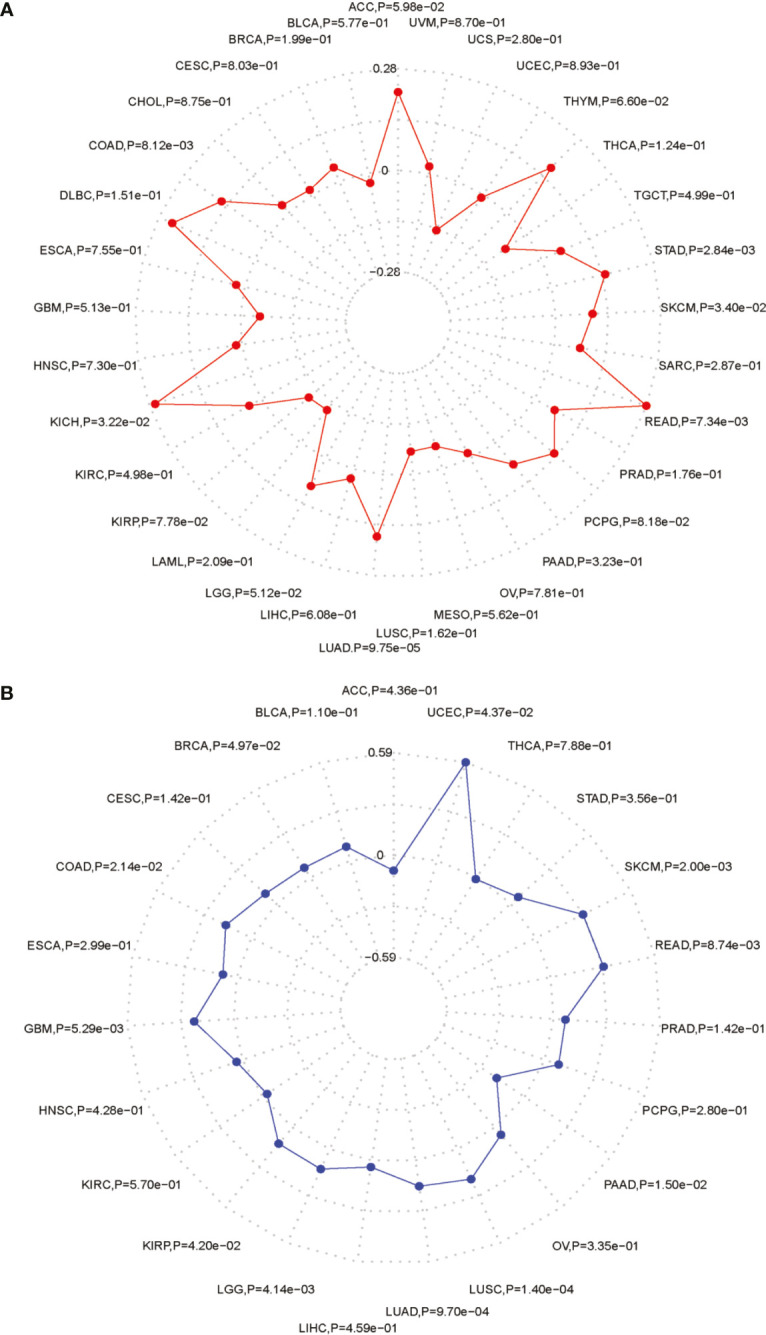
The association between PARP1 expression and TMB levels **(A)**, MSI event **(B)** in tumors.

**Figure 11:**
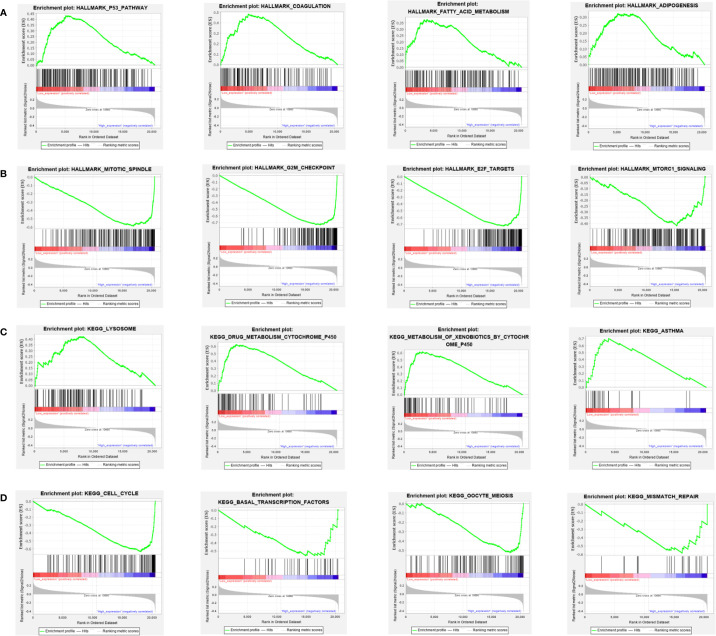
GSEA analysis of HALLMARK **(A, B)** and KEGG **(C, D)** pathway enrichment between PARP1 low expression group and high expression group: the most significant top 4 pathways.

We performed the analysis for the association of PARP1 expression and the number of immune neoantigens, which showed a significant positive association between them around COAD, KIRC, lung adenocarcinoma (LUAD), PAAD and THYM (**Figure 9**), and there were also significant positive correlations between PARP1 expression and TMB in many tumors such as adrenocortical carcinoma (ACC), COAD, kidney chromophobe (KICH), LGG, LUAD, READ, skin cutaneous melanoma (SKCM) and stomach adenocarcinoma (STAD) (**Figure 10A**). Finally, we found that PARP1 expression was closely linked to microsatellite instability event, which suggested that high PARP1 expression was positively associated with microsatellite instability event in COAD, KIRP, BRCA, glioblastoma multiforme (GBM), lung squamous cell carcinoma (LUSC), LGG, READ, UCEC, SKCM and LUAD, while a negative association was observed in PAAD (**Figure 10B**). Finally, in light of the significant positive association between PARP1 expression and the number of immune neoantigens, TMB levels, and MSI in LUAD, we performed enrichment analysis using LUAD transcription data in TCGA by GSEA software, which enrichment analysis demonstrated that the high PARP1 expression group was significantly enriched in KEGG pathways, including CELL CYCLE, and also enriched in HALLMARK terms involving with G2M CHECKPIONT and MTORC1_SIGNALING (**Figure 11**). KEGG_ MISMATCH REPAIR and HALLMARK_ MTORC1_SIGNALING pathway involved in immunotherapy and immunomodulation.

## Discussion

A variety of cancers are driven by genetic alteration, and their genomes contained an average of 4 to 5 driver mutations in combination coding with non-coding genomic elements (20). Another research showed that DNA repair defects could lead to some types of somatic mutation like BRCA1 or 2 (21). However, the role of PARP1 alteration across cancers remained unclear. In our study, we found PARP1 alterations as a variety of nonsynonymous mutations including missense, frameshift, splice site, nonsense, fusions, inframe and deletions, and total alteration rate of PARP1 gene was nearly 3% in 45604 patients with different tumors from TCGA database. Among all these patients, those with skin cancer, non-melanoma had the highest mutation frequency (8.98%, 44/490), followed by patients with endometrial carcinoma (5.29%, 31/586). In addition, our data showed that early-stage tumors were more easily detected by PARP1 amplification in breast invasive carcinomas, liver hepatocellular carcinoma, cholangiocarcinoma, uterine carcinosarcoma, thymoma, lung squamous cell carcinoma, lung adenocarcinoma, esophageal adenocarcinoma, and pancreatic adenocarcinoma, suggesting that PARP1 amplification could be an event of early stage in these tumors such as lung squamous cell carcinoma during cancer evolution (20), while endometrial carcinoma, skin cutaneous melanoma, stomach adenocarcinoma, adrenocortical carcinoma, bladder urothelial carcinoma, colorectal carcinoma, sarcoma, diffuse large-B cell lymphoma, and prostate adenocarcinoma may be easier to detect PARP1 mutations or deep deletions, which is somewhat supported by a prior study reported that multiple mutations typically predated amplification using molecular time analysis (20). Most advanced-stage tumors, including skin cancer, non-melanoma, endometrial cancer, small cell lung cancer, small bowel cancer, gastrointestinal neuroendocrine tumors, and bladder cancer, unlike early-stage tumors, are more prone to arising PARP1 mutations, and a study indicated that PARP1 point mutations were closely linked to *de novo* resistance to PARP1 inhibitors (8), which are usually regarded as synthetic lethal gents in homologous recombination (HR)-deficient tumors, and recent studies have also found that they can prolong progression-free survival in ovarian cancer patients with wild-type BRCA relapse (22, 23) or advanced ovarian cancer patients regardless of BRCA mutation (24), extending their application scope. Therefore, the selection of a suitable subsequent treatment strategy for patients with PARP1 alterations, and their prognostic value, remains unclear. We found the co-occurrence of genetic mutations in tumors with PARP1 alterations to be involved with some genes such as KMT2D/2C as driver genes, PIK3CA, ARID1A, H3F3A, HIST3H3, RYR2, and thus, PARP1 alteration should promote tumor mutability. Therefore, we analyzed the relationship between PARP1 alterations and TMB levels. Surprisingly, the MSK-IMPACT clinical sequencing cohort from the TCGA database (25) showed that TMB levels in the PARP1 altered group were significantly higher than those in the unaltered group, and similar results were verified in two ICI-treated cohorts (26, 27), which indicated that PARP1 alteration could predict ICI treatment effectiveness across tumor types because TMB level is commonly regarded as a notable biomarker associated with the treatment effect in many ICI-treated tumors (27).

We further evaluated the correlation between PARP1 alterations and the clinical prognosis. Patients with PARP1 alterations had markedly longer OS than those with PARP1 unaltered in the whole group and early-stage tumors, and a similar result was found in the ICI-treated cohort. Subgroup analysis revealed that the multiple PARP1 altered group had the best OS compared to those with a single PARP1 altered group. Notably, there was no association between PARP1 alterations and OS/PFS shown in microsatellite-stable (MSS) solid tumors, whereas compared to the PARP1 unaltered group, PARP1 alterations were somewhat linked to better ICIs treatment effect (42.9% *vs.* 27.7%). In early-stage tumors, the PARP1 altered group was closely associated with MSI. The evidence mentioned above suggests that PARP1 alterations might harm MMR and cause tumor phenotype to mutate, or even collaborate with ICI treatment; however, these findings remain to be validated in future studies.

To uncover the potential mechanism of PARP1 alterations that are linked to better ICI treatment effect, we analyzed the relationship between PARP1 alterations and immune infiltrates, including CD8+ T cells, CD4+ T cells, B cells, neutrophils, macrophages, and dendritic cells in all types of tumors from TCGA data by Timer2.0 (15, 28, 29). Impressively, PARP1 mutation was markedly associated with higher levels of immune infiltrates, such as CD8+ T cells, in several tumor types, suggesting that tumors with PARP1 mutations could be classified as active immune subtypes. Meanwhile, the PARP1 alteration group had significantly higher expression of immune checkpoint genes, including LAG3, CTLA4, PDCD1, and TIGHT. These data provide important evidence for PARP1 alterations as pan-cancer predictive biomarkers for ICI treatment. Current research found PARP1 inhibitors (PARPi) remodels tumor immune microenvironment, upregulates PDL1 and drives a systemic Th1-skewing immune response, which activates the priming of immunity and tumor-killing activity, in combination with ICIs for the renaissance of anti-tumor immunity (30), which is somewhat agreement with our results that some patients with PARP1 alterations have an immune-activated microenvironment by promoting tumor mutability, and could boost the sensitivity of patients to ICIs treatment, indicating PARPi and PARP1 alterations might possible represent the similar biological significance to some extent. In addition, although PARP1 alterations like mutations contribute to PARPi resistance (8), our results suggest these partial patients could benefit from ICIs immunotherapy, and another study reported that PARPi combining with chemotherapy, ICIs, alongside with targeted drug had the great advantage in overcoming PARPi resistance as well (31). Therefore, it is necessary to screen the patients with PARP1 alteration for selecting suitable therapeutic strategies.

Moreover, PARP1 alterations were positively associated with high transcription levels of PARP1, which might partially be due to increased PARP1 copy numbers, as evidenced by our results, which is in agreement with results of studies performed on breast cancer (32) and cervical cancer, respectively (33). However, the exact association between PARP1 alteration and its function remain largely unclear, our results showed that PAPR1 altered group, especially in the early-stage tumors, had a better prognosis and the opposite trend is present in advanced-stage tumors. Meanwhile, PAPR1 altered group in the early-stage tumors were predominance of its copy number amplification, and advanced-stage tumors seemed to be predominance of its mutations. Therefore, we speculated, based on those mentioned above, that PAPR1 copy number amplification and its mutations might lead to playing different role in the function of PARP1, thus caused the prognostic difference in early-stage and advanced-stage tumors. We will further explore and verify in future research.

A previous study found that overexpression of BRCA1-associated protein (BRAP) binding to breast cancer suppressor protein (BRCA1) was linked to worse prognosis and immune infiltration in several tumors, and used to as a potential molecular biomarker (34). Therefore, we speculated that DNA damage repair (DDR)-associated gene could have the important biological significance regarding clinical prognosis and treatment. Subsequently, we also analyzed the associations between PARP1 expression and clinical prognosis, immunotherapy signatures. We found that most tumors had high expression of PARP1 mRNA, compared to corresponding normal tissues, and there was a worse prognosis in patients with high expression for several tumors, suggesting that PAPR1 plays an oncogenic role to some extent in multiple tumors, which is consistent with the findings of previous studies on different tumors, such as colorectal cancer (35), gastric cancer (36) and sarcoma (37). Thus patients with high PARP1 expression are required to be monitored with closer follow-up protocols. Another possible reason for PARP1 overexpression is defective PARP1 cleavage, which leads to an imbalance of apoptosis induced by various chemotherapeutic drugs in tumor cells (38, 39), suggesting that these patients with high PARP1 expression might be responsible for chemoresistance. Therefore, whether immunotherapy is suitable for these patients remains to be investigated. We found that high PARP1 expression was significantly associated with six immune cells (B cells, CD4+ T cells, CD8+ T cells, macrophages, neutrophils, and dendritic cells) in most tumors, including COAD, HNSC, KIRC and LIHC etc., indicating that high PARP1 expression group had a greater number of immune cell infiltrations, which is agreement with high PARP1 expression being positively associated with higher immune score and stromal score in KIRC, COAD, UVM, KIRP and READ. In particular, CD8+T cell infiltration, which was also positively correlated with high PARP1 expression in BLCA, BRCA, KIRP, LGG, LIHC, PAAD, PCPG, PRAD, READ, TGCT, THYM, UCEC and UVM. Meanwhile, PARP1 expression was significantly positively correlated with the transcription levels of immune checkpoint genes, such as CD274, CTLA4, and PDCD1, in several tumors including PAAD, LIHC, KIRC, BLCA, and HNSC. These findings mentioned above are similar with a prior study showed that in some tumors, nuclear factor erythroid 2 like 2 (NFE2L2) that is upregulated by transcriptional activation of PARP1 was correlated with immune infiltration and also a potential prognostic biomarker (40, 41). Moreover, there were distinctly negative associations between PARP1 expression and HLA-I and II molecular levels in several tumors. Therefore, we speculated that immune escape may be involved in PARP1-mediated tumorigenesis; thus, immunotherapy such as anti-PDL1/PD1 and/or CTLA4 may be more susceptible in making them have benefits based on these results. Future studies on how to regulate HLA I and II molecular expression that is related to antigen presentation in several tumors with high PARP1 expression, need to be undertaken.

We further performed the analysis for the association of PARP1 expression and the number of immune neoantigens, and a significant positive association were showed between them in COAD, KIRC, LUAD, PAAD and THYM, simultaneously, there were also significant correlations between PARP1 expression and TMB in many tumors, some of which had positive associations including ACC, COAD, KICH, LGG, LUAD, READ, SKCM and STAD. In addition, high PARP1 expression was positively associated with microsatellite instability event in COAD, KIRP, BRCA, GBM, LUSC, LGG, READ, UCEC, SKCM and LUAD, indicating that these tumors with high PARP1 expression could have a better response rate to ICIs immunotherapy, in particular, for LUAD, COAD and READ, LGG with high PARP1 expression, which still needs to be verified in future studies.

In conclusion, the findings of the present study indicate that the pan-cancer analysis of PARP1 alterations and expression were correlated with clinical prognosis, and PARP1 alterations might act as biomarkers in the prediction of immunotherapy effects, and its expression levels seemed to be correlated with the status of immunotherapy-associated signatures, thus they may become promising biomarkers in the prediction of ICI response in several tumors. However, the limitation of this study lies in the need for further experimental verification.

## Data Availability Statement

Publicly available datasets were analyzed in this study. This data can be found here: All the data sets used in this study were publicly available at cBioPortal (https://www.cbioportal.org), TIMER2.0 (http://timer.comp-genomics.org/), and TCGA database (https://xenabrowser.net or https://portal.gdc.cancer.gov/).

## Author Contributions

XZ and JC designed this study. XZ and YW analyzed the data and wrote the manuscript. GA analyzed the data. JC revised the manuscript. All authors contributed to the article and approved the submitted version.

## Funding

This study was supported by the Medical Science Funding of Guangdong province (A2020139), the Youth Foundation of National Natural Science Foundation of China (81902420), and Guangdong Esophageal Cancer Institute Science and Technology Program Project (Q201903).

## Conflict of Interest

The authors declare that the research was conducted in the absence of any commercial or financial relationships that could be construed as a potential conflict of interest.

## Publisher’s Note

All claims expressed in this article are solely those of the authors and do not necessarily represent those of their affiliated organizations, or those of the publisher, the editors and the reviewers. Any product that may be evaluated in this article, or claim that may be made by its manufacturer, is not guaranteed or endorsed by the publisher.

## Supplementary Material

Supplementary Figure S1 The PARP1 alterations rate in early-stage tumors **(A)** and advanced-stage tumors **(B)**.

Supplementary Figure S2 Co-occurrence of genetic mutations in early-stage tumors **(A)** and advanced-stage tumors **(B)** with PARP1 alterations; The association between TMB levels and PARP1 alterations subtypes in MSK-IMPACT cohort **(C)**, 1661 patients with advanced-stage tumors received ICIs-treated cohort **(D)**, 249 patients with microsatellite-stable solid tumors received ICIs treatment **(E)**.

Supplementary Figure S3 Survival analysis of PARP1 alterations and overall survival **(A)**, progress-free survival **(B)** and disease-free survival **(C)** of TCGA cohort; Survival analysis of PARP1 alterations and overall survival **(D)** of MSK-IMPACT cohort.

Supplementary Figure S4 Survival analysis of PARP1 alterations and overall survival **(A)**, progress-free survival **(B)** in patients with microsatellite-stable solid tumors received ICIs treatment.

Supplementary Figure S5 The association of PARP1 alteration and PARP1, immune checkpoints genes expression **(A)**; the association between PARP1 expression and its copy number variations **(B)**; the association between PARP1 copy number variations and immune infiltrates in HNSC **(C)**, KIRC **(D)**, LUAD **(E)**, PAAD **(F)**, SKCM **(G)**, and UCEC **(H)**.
