# Supplementary figures and images for: Pan-Cancer Analysis of PARP1 Alterations as Biomarkers in the Prediction of Immunotherapeutic Effects and the Association of Its Expression Levels and Immunotherapy Signatures
Source: Front Immunol. 2021 Aug 31;12:721030. doi: 10.3389/fimmu.2021.721030 (PMC8438309; doi:10.3389/fimmu.2021.721030)

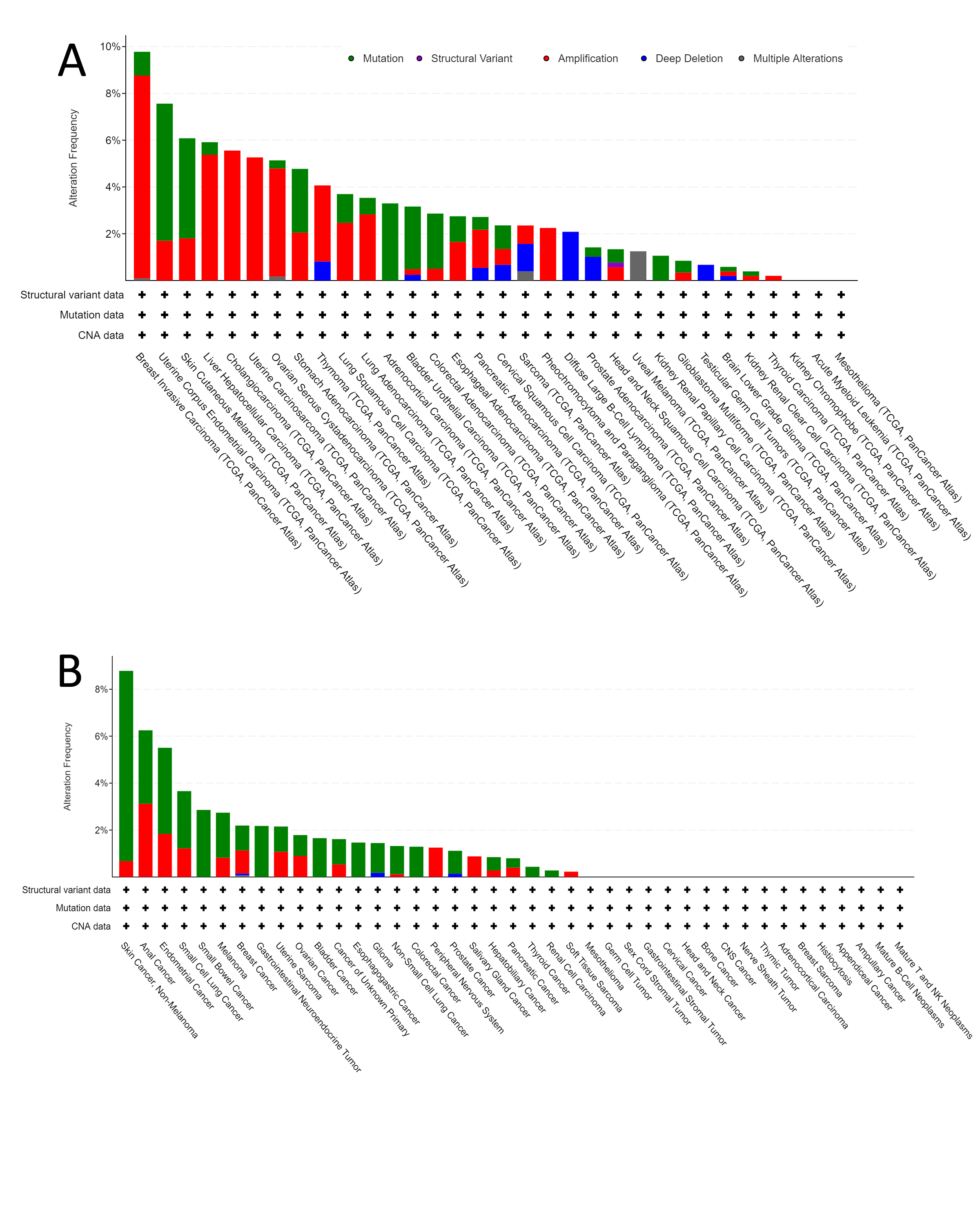

Supplement: Supplementary Figure S1 — The PARP1 alterations rate in early-stage tumors (A) and advanced-stage tumors (B). [file Image_1.tif]

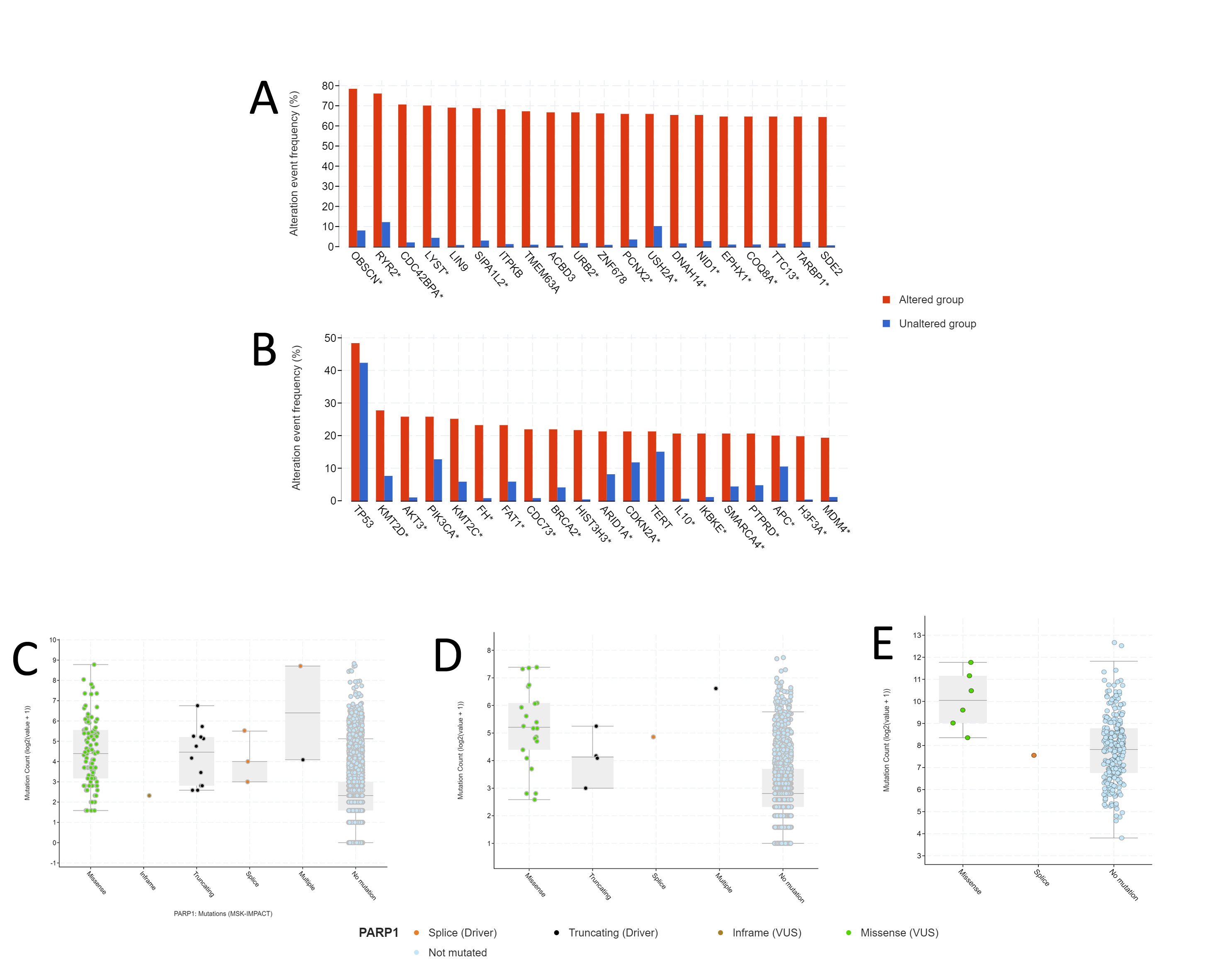

Supplement: Supplementary Figure S2 — Co-occurrence of genetic mutations in early-stage tumors (A) and advanced-stage tumors (B) with PARP1 alterations; The association between TMB levels and PARP1 alterations subtypes in MSK-IMPACT cohort (C), 1661 patients with advanced-stage tumors received ICIs-treated cohort (D), 249 patients with microsatellite-stable solid tumors received ICIs treatment (E). [file Image_2.tif]

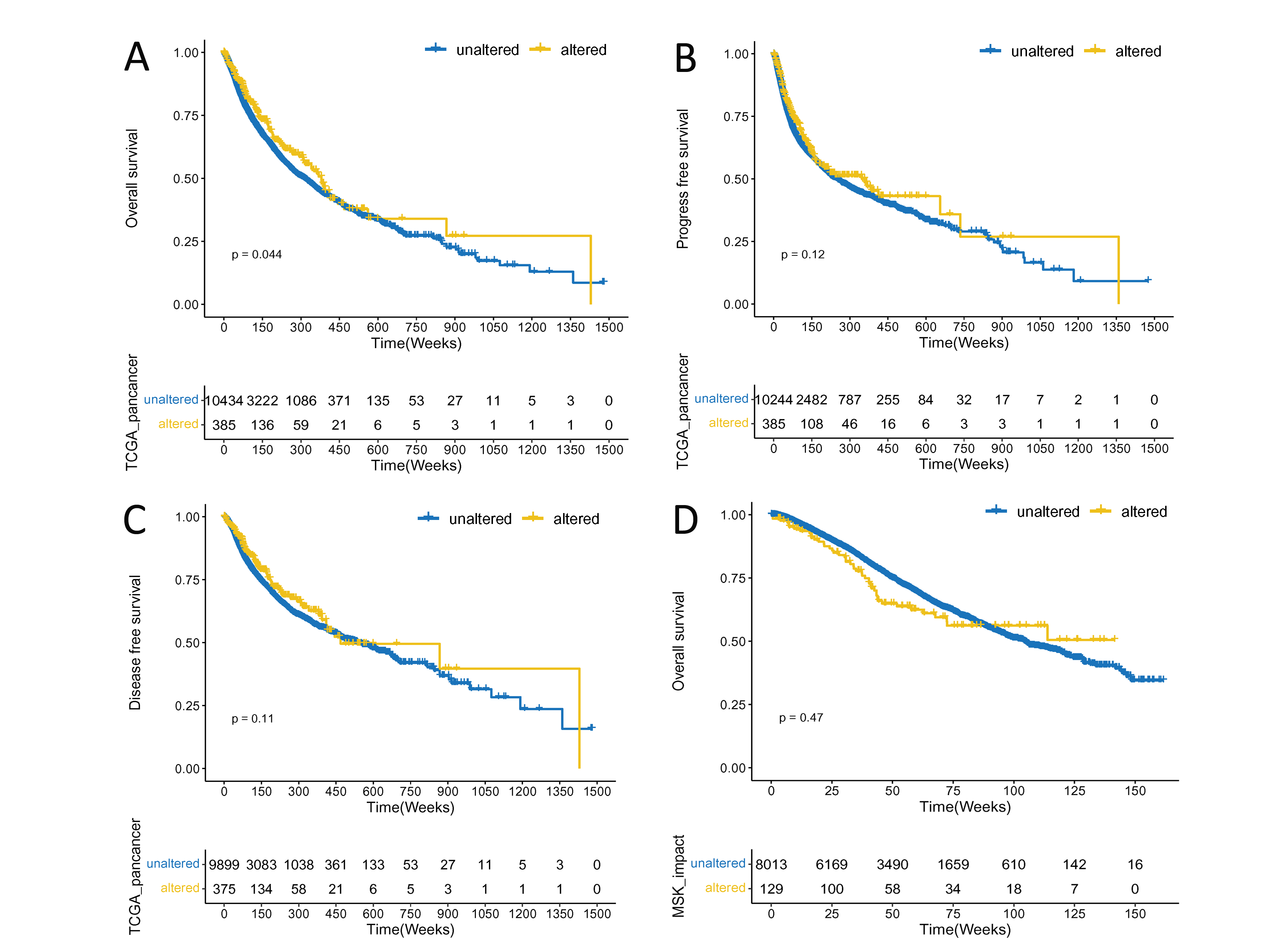

Supplement: Supplementary Figure S3 — Survival analysis of PARP1 alterations and overall survival (A), progress-free survival (B) and disease-free survival (C) of TCGA cohort; Survival analysis of PARP1 alterations and overall survival (D) of MSK-IMPACT cohort. [file Image_3.tif]

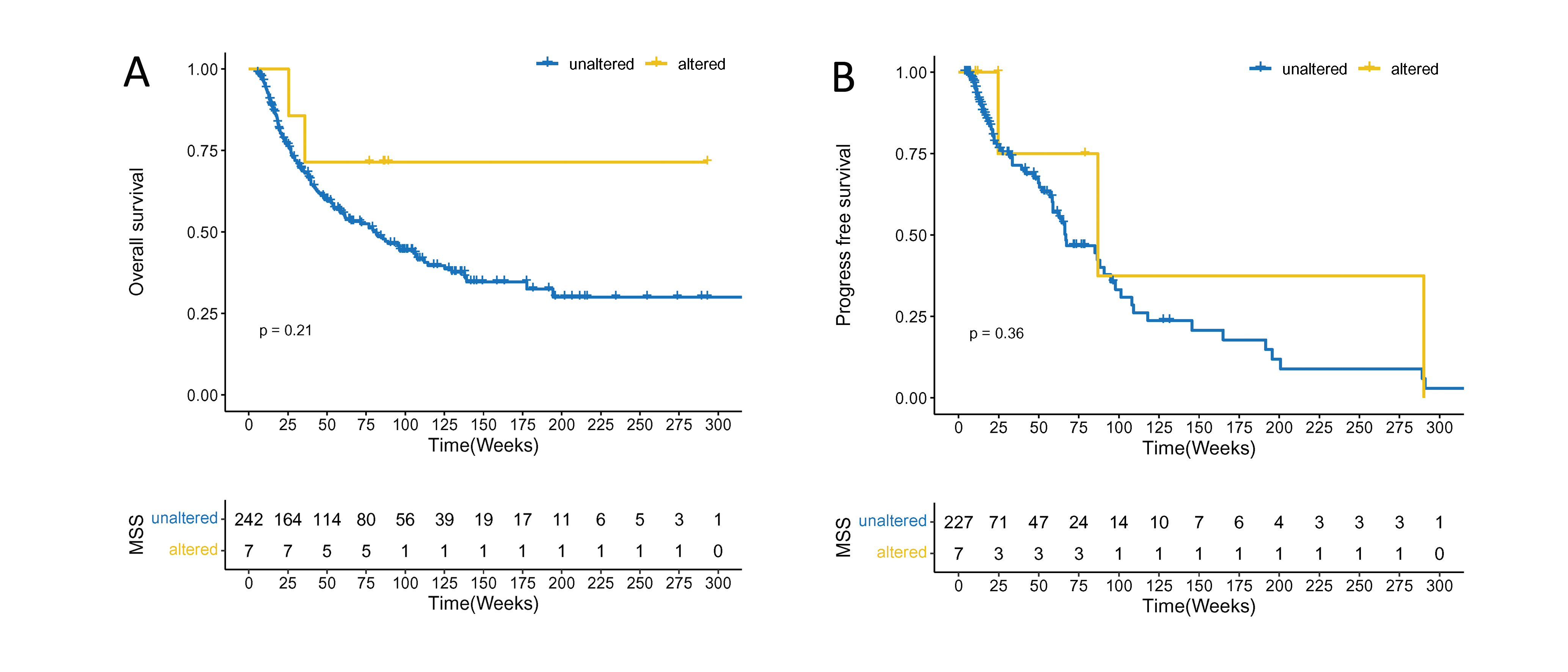

Supplement: Supplementary Figure S4 — Survival analysis of PARP1 alterations and overall survival (A), progress-free survival (B) in patients with microsatellite-stable solid tumors received ICIs treatment. [file Image_4.tif]

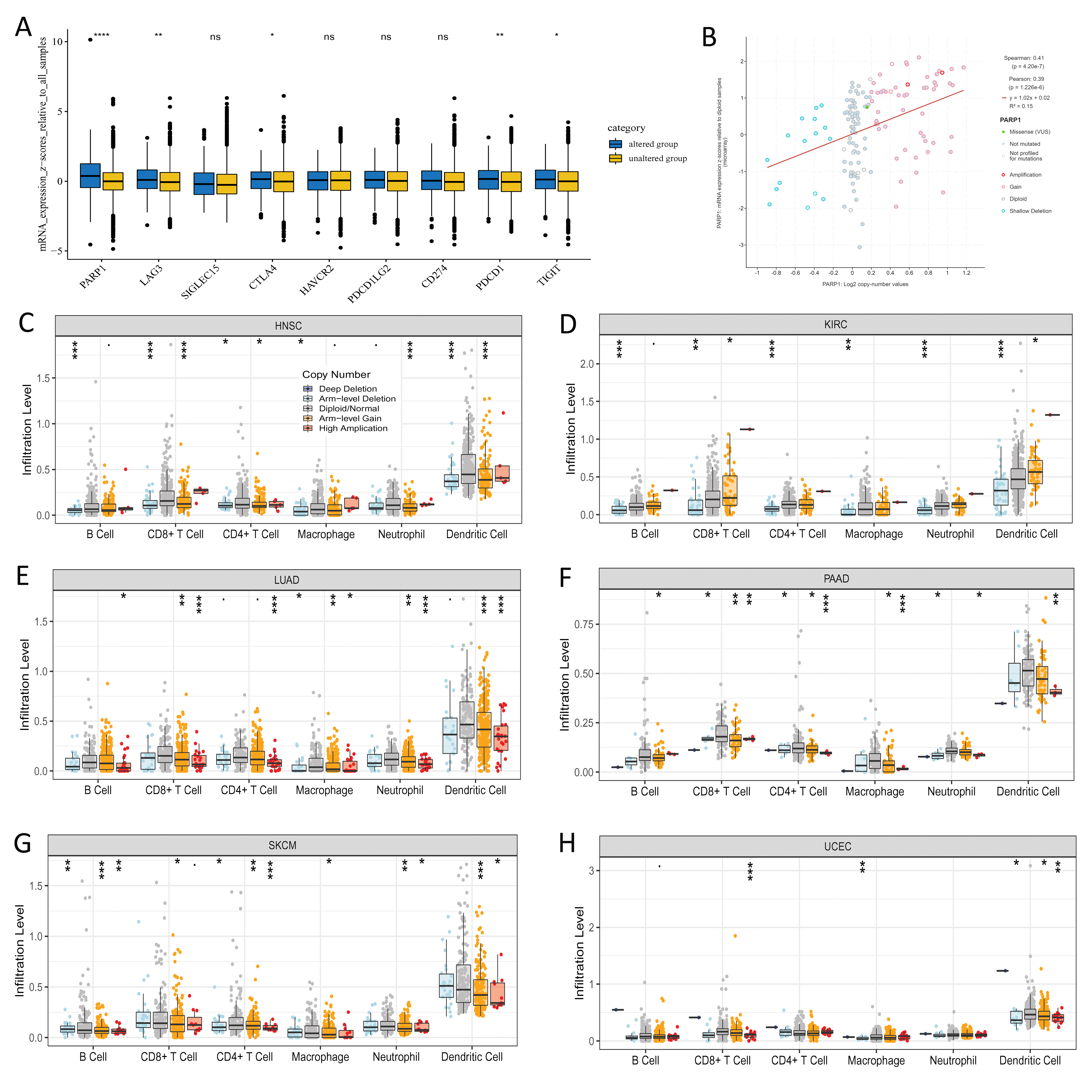

Supplement: Supplementary Figure S5 — The association of PARP1 alteration and PARP1, immune checkpoints genes expression (A); the association between PARP1 expression and its copy number variations (B); the association between PARP1 copy number variations and immune infiltrates in HNSC (C), KIRC (D), LUAD (E), PAAD (F), SKCM (G), and UCEC (H). [file Image_5.tif]
